# Impact of Roasting on Functional Properties of Hard-to-Cook Beans Under Adverse Storage Conditions

**DOI:** 10.3390/foods14030470

**Published:** 2025-02-01

**Authors:** Takako Koriyama, Kiriko Teranaka, Michiyo Kumagai

**Affiliations:** 1 Faculty of Food and Nutritional Science, Toyo University, 48-1 Oka, Asaka-shi, Saitama 351-8510, Japan; 2 Department of Nutrition and Food Science, Graduate School of Humanities and Science, Ochanomizu University, 2-1-1 Otsuka, Bunkyo-ku, Tokyo 112-8610, Japan; 3 Department of Food Sciences, Tokyo Seiei College, 1-4-6 Nishishinkoiwa, Katsushika-ku, Tokyo 124-8530, Japan; kumagai-m@tsc-05.ac.jp

**Keywords:** hard-to-cook beans, roasting, antioxidant activity, resistant starch, functional properties

## Abstract

This study examined the responses of four legumes—chickpeas (*Cicer arietinum* L.), red kidney beans (*Phaseolus vulgaris* L., Taishokintoki), adzuki beans (*Vigna angularis*), and peanuts (*Arachis hypogaea*)—to storage and roasting under high-temperature and high-humidity conditions (HTC beans). Roasting enhanced antioxidant activity in HTC chickpeas and peanuts, with chickpeas also showing increased resistant starch. In contrast, kidney beans showed reduced resistant starch after storage, with minimal recovery upon roasting, while refrigeration better preserved resistant starch. For adzuki beans, roasting reduced resistant starch in control samples but not in HTC samples. Reducing sugars decreased in all beans after roasting. These findings highlight roasting as a promising method for repurposing HTC chickpeas and peanuts for functional food applications. Limitations include variability among legumes and the need for further mechanistic and sensory studies.

## 1. Introduction

Legumes are a valuable food source that are rich in essential nutrients and functional components. Typically, legumes are stored for a period before consumption. However, when stored under unfavorable conditions, such as high temperature and high humidity, softening them becomes challenging, even with extended cooking. This issue, referred to as “hardening” or the “hard-to-cook (HTC)” defect, poses significant challenges in areas where legumes represent a dietary staple [1]. The causes of the hardening of beans have been reported to be the insolubilization of acidic and neutral pectins in the cell walls, ligninization of the cell walls, and inhibition of starch swelling and gelatinization due to the denaturation of proteins surrounding starch [2,3]. Even in beans that contain little starch, such as soybeans, hardening progresses when stored under high-temperature and high-humidity conditions, and the denaturation of cotyledon proteins has been suggested as one of the causes of hardening [4]. The HTC phenomenon reduces both the palatability and bioavailability of the legumes, leading to their disposal. Despite these challenges, research on effective processing methods for hardened legumes remains limited [5]. Considering that the hardened legumes are usually discarded, it is crucial to explore new ways to utilize them. The HTC phenomenon poses a significant technological challenge but also highlights the potential for the development of sustainable food processing solutions. By developing effective processing techniques, such as roasting, the food industry can convert waste into valuable products, in turn minimizing adverse environmental impacts while facilitating meeting the demand for functional foods by consumers. Roasting is a heat-based process known to enhance the antioxidant properties of foods by generating Maillard reaction products (MRPs). The Maillard reaction, which occurs between reducing sugars and amino acids during roasting, not only enhances flavor profiles but also contributes to the formation of bioactive compounds, such as melanoidins, which are known for their antioxidant properties [3,6]. For example, roasted soybean flour (kinako) is known for its strong antioxidant activity, attributed to Maillard reaction products formed during roasting [3,6]. Roasting enhances the antioxidant capacity of various foods, including sesame seeds, nuts, and corn [7,8,9]. Based on this evidence, we hypothesized that roasting could improve the quality of hardened beans that have undergone prolonged storage under adverse conditions.

In our previous study, we investigated the effects of storage and roasting on protein-rich soybeans with yellow, blue, and black seed coats stored under high-temperature and high-humidity conditions (30 °C, 75% RH) for 60 days. The findings revealed a slight increase in antioxidant activity in yellow soybeans after storage, whereas blue and black soybeans showed a slight decrease. However, roasting, irrespective of seed coat color, led to a significant increase in the antioxidant activity of all soybean types [10]. Additionally, roasting not only improved the flavor and functionality of hardened soybeans, but also made them easier to grind into powder. The possibility of processing into powder is advantageous for food production, e.g., when making bread and noodles [11,12]. These findings suggest that roasting could be a promising method for utilizing hardened legumes.

Legumes comprise a wide range of nutritional and functional components. For example, chickpeas (*Cicer arietinum* L.), red kidney beans (*Phaseolus vulgaris* L.), adzuki beans (*Vigna angularis*), and peanuts (*Arachis hypogaea*) are rich in functional components, such as antioxidants, resistant starch, and reducing sugars, which contribute to their health benefits. The compositions of these legumes change during storage [13,14]. We observed the hardening phenomenon in various legumes, such as common, adzuki, red kidney, white kidney, black, and Mucuna beans as well as chickpeas stored in an environment with elevated temperature and humidity [10]. These legumes showed varying degrees of reduced water absorption and softening rates, indicating differences in the extent of hardening, which suggests that the impact of roasting on hardened legumes may vary depending on their composition.

The present study aimed to evaluate whether roasting could enhance the functionality of legumes with diverse compositions after storage under high-temperature and high-humidity conditions. Specifically, we sought to clarify the impact of storage conditions and roasting on antioxidant activity, reducing sugar content, and resistant starch content in four types of legumes—chickpeas (*Cicer arietinum* L.), red kidney beans (*Phaseolus vulgaris* L., Taishokintoki), adzuki beans (*Vigna angularis*), and peanuts (*Arachis hypogaea*).

## 2. Materials and Methods

### 2.1. Plant Material and Storage Conditions

Samples of chickpeas, red kidney beans, adzuki beans, and peanuts, all of which were newly harvested in Japan, were purchased from an online specialty bean store and stored in sealed plastic bags at 4 °C and 80% RH (under which no hardening occurs) until use [10]. As peanuts belong to the Fabaceae family, they were included among the samples [15]. HTC beans were prepared by storing samples in a thermostatic chamber (MOV-212S, Sanyo, Osaka, Japan), set at 30 °C ± 1 °C and 75% ± 3% RH for 60 d, conditions commonly used to simulate subtropical storage environments. The parameters were selected based on previous studies that have reported significant effects of the conditions on the properties of legumes [2,10,14]; in addition, they reflect typical ambient conditions during summer in Japan, where legumes are often stored at room temperature. The temperature and relative humidity in the thermostatic chamber were monitored continuously and maintained using calibrated sensors, ensuring precise and stable conditions throughout the experiment. Visually inspected beans were screened to remove broken or immature samples before the experiment.

### 2.2. General Ingredients of Beans

The general composition of each legume was analyzed using near-infrared spectroscopy with the Calorie Answer^™^ system (CA-Hi, JWP, Aomori, Japan). Reflectance mode was employed in combination with the Beans mode for accurate analysis. Each sample was scanned five times, and the average spectrum was processed using CA-HM Measurement Application Software (JWP, Aomori, Japan) to ensure accuracy.

### 2.3. Measurement of Moisture Content

The moisture content of the beans was measured using the AOAC atmospheric drying method [16]. A 2 g sample was placed in a pre-weighed aluminum dish (*W*_0_) and heated at 135 °C for 4 h until a constant weight (*W*_2_) was achieved. The moisture content was calculated based on the weight loss using Equation (1):

(1) $$Moisture\;Content\;\left(\%\right)=\frac{\left(W_{1}-W_{2}\right)}{\left(W_{1}-W_{0}\right)}\times 100$$

Reducing the moisture content of beans to below 18% prior to storage is critical for maintaining their safety and quality [5]. In the present study, the initial moisture content of the stored beans ranged from 6.1% to 14.8%. After storage under high-temperature and high-humidity conditions, the moisture contents were 11.2%, 15.5%, 14.7%, and 6.3% for chickpeas, kidney beans, adzuki beans, and peanuts, respectively, indicating no significant differences in moisture content before and after storage. Hence, the storage conditions employed in the present study were not conducive to the growth of fungi, as no signs of fungal contamination were observed during the experimental period.

In large-scale storage systems, particularly under elevated moisture content conditions, regular monitoring for potential infestations, such as stored insects or fungal contamination (e.g., *Aspergillus candidus*), is essential. Weekly inspections are recommended to identify and mitigate any potential quality degradation during storage. Although the present study used controlled and sealed storage conditions that minimized contamination risks, the importance of periodic assessments in practical storage environments cannot be overlooked.

### 2.4. Roasting Conditions

Approximately 50 g of each bean type was evenly distributed on a baking sheet (to prevent overlap) and roasted for 20 min in a gas oven set at 190 °C. This temperature was selected based on the results of our previous study [17,18], which demonstrated that roasting legumes at temperatures ranging from 150 to 210 °C enhances antioxidant activity and promotes Maillard reaction product formation, contributing to improved functionality. Additionally, 190 °C represents a balance between achieving uniform browning and avoiding excessive burning, ensuring practical applicability in food processing.

### 2.5. Measurement of Color

Color values were measured using a spectrocolorimeter (CM-700d, Konica Minolta, Tokyo, Japan) and expressed in terms of L*, a*, and b*, according to the CIE color scale. L* represents brightness ranging from 0 (black) to 100 (white), a* indicates the degree of redness (+a*) or greenness (−a*), and b* indicates yellowness (+b*) or blueness (−b*). The color difference (Δ*E*) caused by roasting was calculated using Equation (2):

(2) $$\Delta E={\;[{\left(L^{*}Roasted-L^{*}Unroasted,4{}^{\circ}C,\;80\%\;RH\right)}^{2}+{\left(a^{*}Roasted-\;a^{*}Unroasted,4{}^{\circ}C,\;80\%\;RH\right)}^{2}+{\left(b^{*}Roasted-b^{*}Unroasted,4{}^{\circ}C,\;80\%\;RH\right)}^{2}]}^{1/2}$$

The total color change (ΔE) was calculated as the difference in color values relative to the unroasted beans stored at 4 °C and 80% RH. The experiment was conducted in triplicate.

### 2.6. Measurement of Antioxidant Activity

To prepare the extract for antioxidant activity assays, 1 g of bean powder was mixed with 50 mL of 80% ethanol and shaken at 136 rpm for 60 min at 25 °C. The mixture was then centrifuged at 11,070× *g* for 15 min at 4 °C, and the supernatant was filtered through a 0.45 μm membrane filter. This extract was subsequently analyzed using the 1,1-diphenyl-2-picrylhydrazyl (DPPH) free radical scavenging assay and the oxygen radical absorbance capacity (ORAC) method.

The DPPH free radical scavenging activity was measured according to the method of Blois [19]. After appropriate dilution, 50 μL of the sample was mixed with 0.4 mL of ethanol, 50 μL of 0.1 M acetate buffer (pH 5.5), and 100 μL of 0.5 mM DPPH reagent. The mixture was stored in the dark at 20 °C for 20 min, and absorbance was measured at 517 nm using a spectrophotometer (Synergy HTX, Biotek, Winooski, VT, USA). The results were expressed as micromoles of Trolox equivalents per gram of legume (µmol TE/g) on a dry weight basis, using a Trolox calibration curve. All measurements were performed in triplicate.

The ORAC assay was conducted as described by Huang et al. [20]. A 25 μL diluted sample, prepared with 75 mM phosphate buffer, was placed in a black 96-well microplate, followed by the addition of 150 μL of 86.1 nM fluorescein. After incubation at 37 °C for 10 min, 50 μL of a 320 mM 2,2-azobis(2-amidinopropane) dihydrochloride (AAPH) solution was added, and fluorescence decay was monitored at 760 nm using a spectrophotometer (Synergy HTX, Biotek, USA). ORAC values were expressed in µmol TE/g on a dry weight basis using a Trolox calibration curve (linear range: 5.0–50 µM). All experiments were conducted in triplicate.

### 2.7. Measurement of Total Phenolic Content

The Folin–Ciocalteu method, with tannic acid as the standard, was used to measure the total phenolic content (TPC) [21]. For extraction, 1 g of legume powder was mixed with 50 mL of 80% methanol and shaken at 136 rpm for 60 min at 25 °C. The mixture was centrifuged at 11,070× *g* for 15 min at 4 °C, and the supernatant was filtered through a 0.45 μm membrane filter. To prepare the reaction mixture, 0.5 mL of the extract was combined with 0.5 mL of distilled water, 0.1 mL of 4-fold-diluted Folin–Ciocalteu reagent (Fujifilm Wako Pure Chemical Corporation, Tokyo, Japan), and 0.2 mL of 10% Na_2_CO_3_ solution. After allowing the mixture to stand at 25 °C for 60 min, absorbance was measured at 760 nm using a spectrophotometer (Synergy HTX, Biotek, USA). The TPC was determined using a tannic acid calibration curve and expressed as mg/g of dry weight. All experiments were performed in triplicate.

### 2.8. Measurement of Reducing Sugar Content

The reducing sugar content was measured using the Somogyi–Nelson method [22,23], with glucose as the standard for quantifying total and reducing sugars. Reagent C (alkaline copper solution) and reagent D (arsenomolybdate solution) were prepared according to the Somogyi–Nelson protocol [22]. All chemicals used were of analytical grade. Briefly, 0.3 mL of the sample and 0.3 mL of reagent C were added to a test tube, following which it was sealed and heated in a boiling water bath for 15 min. After cooling to 25 °C, 0.3 mL of reagent D was added to it, followed by vortexing. Then, after adding 0.9 mL of reverse osmosis water, the mixture was vortexed again, and the absorbance was measured at 520 nm using a spectrophotometer. All measurements were performed in triplicate.

### 2.9. Measurement of Resistant Starch Content

The resistant starch content was measured using the Megazyme Resistant Starch Assay Kit (K-RSTAR, Megazyme International, Wicklow, Ireland) according to the manufacturer’s instructions and the method described by McCleary and Monaghan [24]. Briefly, samples were treated with pancreatic α-amylase and amyloglucosidase at 37 °C for 16 h to hydrolyze digestible starch. After centrifugation (1500× *g*, 10 min, 4 °C) and subsequent washing with ethanol, the resistant starch was solubilized with KOH, and glucose was released after treatment with amyloglucosidase. The glucose content was determined using the GOPOD reagent, with the absorbance measured at 510 nm using a spectrophotometer. The resistant starch content was calculated based on the glucose concentration. All analyses were performed in triplicate.

### 2.10. Statistical Analyses

Data were analyzed using SPSS version 28.0 for Windows (IBM Corp., Armonk, NY, USA). The results are presented as the mean ± standard deviation. An unpaired *t*-test was used to compare color values, while one-way analysis of variance was used to assess differences in antioxidant activity, TPC, reducing sugar content, and color. When significant differences among samples were identified (*p* < 0.05), Tukey’s honestly significant difference test was applied for post hoc multiple comparisons. Effects were considered statistically significant if *p* < 0.05 or *p* < 0.01.

## 3. Results and Discussion

### 3.1. Nutritional Composition and Storage Safety of Legumes

The nutritional composition of chickpeas, red kidney beans, adzuki beans, and peanuts is summarized in Table 1. Peanuts had the highest energy content (545 ± 12.7 kcal/100 g), primarily due to their high lipid content (47.3 ± 0.71 g/100 g). In contrast, chickpeas showed the highest carbohydrate content (62.3 ± 0.53 g/100 g). Protein levels were relatively high in red kidney beans (22.6 ± 0.14 g/100 g) and peanuts (24.2 ± 0.09 g/100 g). Sodium levels were negligible in all samples. These results highlight the nutritional diversity among legumes and their potential as functional food ingredients.

Reducing the moisture content of beans to below 18% prior to storage is critical for maintaining their safety and quality [5]. In the present study, the initial moisture content of the stored beans ranged from 6.1% to 14.8%. After storage under high-temperature and high-humidity conditions, the moisture contents were 11.2%, 15.5%, 14.7%, and 6.3% for chickpeas, kidney beans, adzuki beans, and peanuts, respectively, indicating no significant differences in moisture content before and after storage. Hence, the storage conditions employed in the present study were not conducive to the growth of fungi, as no signs of fungal contamination were observed during the experimental period.

### 3.2. Changes in the Appearance and Color of Beans

Table 2 presents changes in color values for various kinds of beans following storage and roasting. The beans stored at 4 °C and 80% RH served as the control group, which showed no significant changes during storage, whereas the beans stored at 30 °C and 75% RH for 60 d were designated as HTC beans. The difference in color values between the control and HTC beans in the unroasted state reflects the impact of storage conditions, whereas the difference between unroasted and roasted samples of both the control and HTC beans indicates the impact of roasting.

Unroasted HTC beans exhibited a significant decrease in the L* value (lightness) and an increase in the a* value (red–green scale) compared with the unroasted control beans. This darkening effect can be attributed to the degradation of natural pigments and accumulation of intermediate products such as reducing sugars and amino acids during storage under high-temperature and high-humidity conditions. Such conditions likely accelerated oxidative reactions and promoted non-enzymatic browning, which laid the foundation for the enhanced browning observed during subsequent roasting. Among the bean types, chickpeas and peanuts showed the most pronounced changes in L* and a* values during storage, suggesting that their chemical composition, including their higher levels of reducing sugars and reactive amino acids, might predispose them to greater color changes.

Roasting induced significant color changes in all bean types, with marked reductions in L* values and increases in a* and b* (yellow–blue scale) values. The changes were most evident in roasted HTC beans compared with their control counterparts. The more pronounced browning of HTC beans during roasting indicates a synergistic effect of storage and roasting. Specifically, storage under high-temperature and high-humidity conditions likely increased the availability of reducing sugars and amino acids, which serve as substrates for the Maillard reaction during roasting. This reaction is a major contributor to the formation of brown pigments and thus the observed darkening of the beans. Notably, peanuts exhibited the greatest decrease in L* values during roasting, possibly due to their higher lipid content, which could enhance thermal degradation and promote browning through lipid oxidation and interaction with Maillard reaction intermediates. Adzuki beans and red kidney beans, conversely, demonstrated relatively stable b* values despite significant increases in a* values, suggesting that the development of red hues might overshadow yellow hues in these legumes.

The ΔE values (total color difference) underscore the combined effects of storage and roasting, which were most pronounced in HTC beans. This suggests that high-temperature and high-humidity storage not only alters the initial color properties of beans but also enhances their susceptibility to browning during roasting. The findings highlight the importance of controlling storage conditions to modulate the color attributes of roasted beans, particularly in applications where uniformity in color is a critical quality parameter.

The differences among bean types in their responses to storage and roasting reflect variations in composition, including levels of reducing sugars, amino acids, and seed coat pigments. Chickpeas, with their lighter initial color and higher protein content, exhibited significant shifts in a* and b* values, likely due to intense Maillard reactions [25,26]. Conversely, adzuki beans, characterized by their naturally darker seed coat and anthocyanin content, exhibited relatively smaller changes in L* values but notable increases in a* values, indicative of enhanced red pigmentation. In conclusion, high-temperature and high-humidity storage conditions not only directly affect bean color but also intensify the effects of roasting. The findings provide important insights for optimizing the processing of legumes, particularly in the context of developing food products with desirable visual and sensory attributes.

### 3.3. Changes in the Antioxidant Activity and TPC of Beans

**Figure 1:**
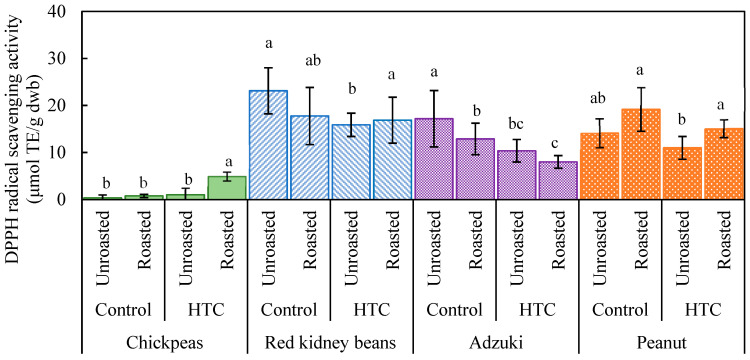
1,1-Diphenyl-2-picrylhydrazyl (DPPH) radical scavenging activity of various beans as affected by storage conditions and roasting. Controls were stored at 4 °C and 80% relative humidity (RH) for 60 d; hard-to-cook (HTC) beans were stored at 30 °C and 75% RH for 60 d. Roasting was performed at 190 °C for 20 min. Significant differences within the same bean type are indicated by lowercase letters a, b, and c. *p* < 0.05 (n = 3).

Figure 1 illustrates the DPPH radical scavenging activity of various beans, both roasted and unroasted. In chickpeas, the activity was very low, except in the case of the roasted HTC beans, indicating that DPPH radical scavenging activity emerged only after both storage and roasting. Antioxidant activity was significantly higher in red kidney beans and adzuki beans than in chickpeas, but this activity decreased notably after storage. The DPPH radical scavenging activity of adzuki beans in both the control and HTC groups decreased following roasting, whereas that of red kidney beans remained stable. Generally, darker-colored foods have higher antioxidant activity. In the current study, colored beans, such as kidney beans and adzuki beans, had higher antioxidant activity than chickpeas, likely owing to the presence of different types of pigments. Specifically, chickpeas contain flavonoids, red kidney beans contain anthocyanins, and adzuki beans contain procyanidins [27]. In peanuts, the outer skin, which contains the antioxidant luteolin, contributes significantly to antioxidant activity [28].

**Figure 2:**
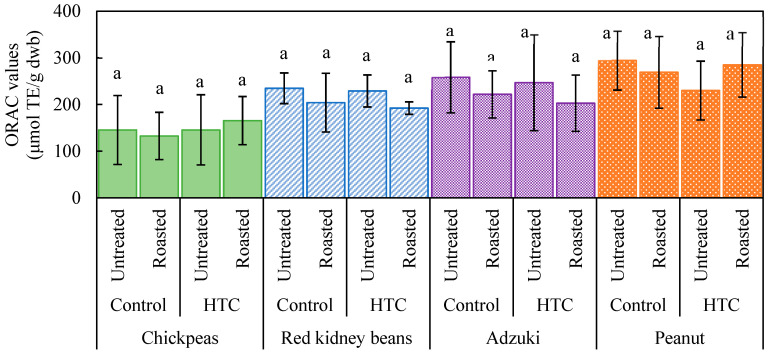
Oxygen radical absorbance capacity (ORAC) values of various beans as affected by storage conditions and roasting. Controls were stored at 4 °C and 80% relative humidity (RH) for 60 d; hard-to-cook (HTC) beans were stored at 30 °C and 75% RH for 60 d. Roasting was performed at 190 °C for 20 min. No significant differences were observed within the same bean type, as indicated by the same lowercase letter “a”. *p* < 0.05 (n = 3).

The effects of storage conditions and roasting on antioxidant activity varied across different types of beans. As shown in Figure 1, based on DPPH radical scavenging activity in both the control and HTC kidney beans, the antioxidant activity did not change significantly after roasting, although a decrease was noted following storage. For adzuki beans, both storage conditions and roasting decreased antioxidant activity. For peanuts, no clear differences were observed between the control and HTC beans when unroasted; however, antioxidant activity increased after roasting for both groups. These results suggest that roasting enhances the antioxidant activity of both chickpeas and peanuts. Conversely, kidney beans and adzuki beans stored under high-temperature and high-humidity conditions showed reduced antioxidant activity. However, both kidney beans and adzuki beans retained higher antioxidant activity than chickpeas, indicating that they contained higher levels of antioxidant compounds and could retain a significant level of antioxidant activity even after roasting. In contrast, as shown in Figure 2, the ORAC assay results indicated no significant differences across all the beans, suggesting divergence in the trends observed with the two antioxidant assays. The findings highlight that the two methods measure different aspects of antioxidant activity, which may be influenced by the chemical composition of the beans and their responses to processing.

**Figure 3:**
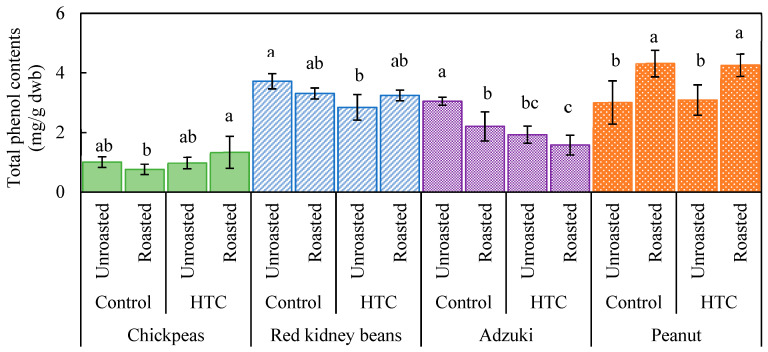
Total phenolic content of various beans as affected by storage conditions and roasting. Controls were stored at 4 °C and 80% relative humidity (RH) for 60 d; hard-to-cook (HTC) beans were stored at 30 °C and 75% RH for 60 d. Roasting was performed at 190 °C for 20 min. Significant differences within the same bean type are indicated by lowercase letters a, b, and c. *p* < 0.05 (n = 3).

The TPC of different beans is shown in Figure 3. Chickpeas showed the lowest TPC, which remained relatively unchanged after both storage and roasting, although roasting slightly increased the TPC in HTC beans. For both control and HTC red kidney beans, the TPC did not significantly change after roasting. However, the TPC decreased significantly in the HTC samples when compared with that in the control samples, consistent with the trends observed for other legumes. For adzuki beans, the TPC decreased following both storage and roasting, with roasted HTC beans showing a reduction of up to 50% compared with the control beans. No clear differences in the TPC were observed between the control and HTC unroasted peanuts; however, roasting significantly increased the TPC. These findings suggest that storage under high-temperature and high-humidity conditions has minimal impact on the TPC of chickpeas and peanuts, but significantly reduces the TPC in kidney beans and adzuki beans.

A comparison of Figure 1 and Figure 3 illustrates the correlation between DPPH radical scavenging activity and the TPC, particularly for kidney beans and adzuki beans. This correlation suggests that phenolic compounds in the seed coat of these beans may become more susceptible to oxidation and degradation under high-temperature and high-humidity storage conditions, leading to a reduction in antioxidant components [29]. Machado et al. [30] reported that kidney beans with different seed coat colors (white, red, or black) showed reduced antioxidant capacity when hardened owing to high-temperature and high-humidity storage, with a strong correlation between antioxidant activity and the TPC. We previously noted that soybeans with different seed coat colors responded differently to high-temperature and high-humidity storage—antioxidant activity and the TPC increased in yellow soybeans, but decreased in blue and black soybeans [17]. This reduction was likely owing to the loss of anthocyanin-based phenolic compounds, which are abundant in the outer skins of these beans. Konishi et al. [18] reported that roasting adzuki beans at 160 °C for 15 min resulted in a significant decrease in both DPPH radical scavenging activity (77.6%) and the TPC (67.8%; *p* < 0.05), attributing this decrease to the thermal degradation of polyphenols. In contrast, peanuts and chickpeas showed an increase in both the TPC and DPPH radical scavenging activity after roasting. This suggests that roasting may generate additional antioxidant components in chickpeas and peanuts, such as flavonoids and other antioxidants, which enhance the overall antioxidant activity [31]. Furthermore, heat treatment may damage cell walls, facilitating the release of antioxidant compounds, such as phenolics, and improving extraction efficiency. The antioxidant activity of peanuts is associated with flavonoids, protocatechuic acid ethyl esters, tocopherols, and resveratrol [28,32]. The DPPH radical scavenging activity of peanuts decreased after storage at 35 °C for 2 months [33]. Peanuts contain many lipid components that are prone to oxidation during roasting or storage; therefore, antioxidant components preventing such oxidation are likely depleted during these processes. However, in the current study, no significant differences were observed between the control and HTC peanut groups, and roasting was found to increase the antioxidant activity of peanuts. This finding indicates that roasting can potentially enhance the antioxidant activity of peanuts, regardless of storage conditions.

Although kidney beans and adzuki beans showed decreases in the TPC and DPPH radical scavenging activity under high-temperature and high-humidity storage, their inherently high antioxidant levels allowed them to retain a certain degree of antioxidant activity even after roasting. In contrast, chickpeas in the control group showed low antioxidant activity, which was less affected by storage conditions and roasting. However, the antioxidant activity of HTC beans significantly increased after roasting.

Regarding the effective processing of HTC beans for antioxidant activity, the results suggested that adzuki beans could be used without roasting, whereas for HTC chickpeas and peanuts, roasting could provide added value. When roasted and powdered, these beans can be used in various culinary applications as functional ingredients, potentially replacing other powders. Based on our findings from the current and previous study, we recommend roasting as a method to enhance the antioxidant activity of soybeans, chickpeas, and peanuts.

### 3.4. Changes in the Reducing Sugar Content of Beans

**Figure 4:**
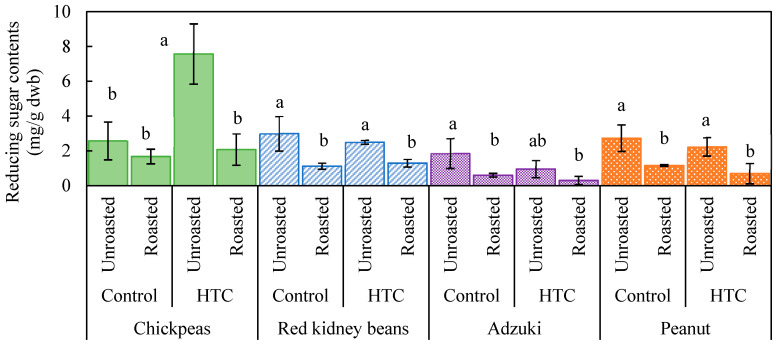
Reducing sugar content of various beans as affected by storage conditions and roasting. Controls were stored at 4 °C and 80% relative humidity (RH) for 60 d; hard-to-cook (HTC) beans were stored at 30 °C and 75% RH for 60 d. Roasting was performed at 190 °C for 20 min. Significant differences within the same bean type are indicated by lowercase letters a and b. *p* < 0.05 (n = 3).

Figure 4 shows changes in the reducing sugar content of different beans after storage and roasting. Significant differences were observed only for chickpeas, in which the reducing sugar content of the HTC beans was approximately four times higher than that of the control group. This increase suggests that the enzymatic breakdown of starch contributed to the formation of reducing sugars under high-temperature and high-humidity storage conditions. Chickpeas, which have relatively higher amylose contents than other beans [34,35], may exhibit greater susceptibility to enzymatic hydrolysis under such conditions. The reducing sugar contents decreased in all beans after roasting, likely due to the partial thermal degradation of reducing sugars and their consumption through the Maillard reaction. The Maillard reaction not only decreases reducing sugars but also produces Maillard reaction products that are known to have antioxidative properties. This was evident in chickpeas and peanuts, where the reduction in the reducing sugar content corresponded to an increase in antioxidant activity (Figure 1).

For kidney beans and adzuki beans, the antioxidant activity after roasting might have been influenced by factors other than the Maillard reaction, such as the degradation of polyphenols or the release of bound phenolic compounds. The results highlight the complexity of changes in functional parameters during roasting, emphasizing the need for further studies to isolate and identify the key compounds responsible for antioxidant activity in each type of bean. Chickpeas have relatively higher amylose contents than other beans [34,35]. In the present study, reducing sugar contents decreased in all beans after roasting, likely owing to the partial thermal degradation of reducing sugars or their consumption via the Maillard reaction during roasting. This decrease in reducing sugars in chickpeas and peanuts corresponded to an increase in antioxidant activity (Figure 1), suggesting that the Maillard reaction contributed to the observed rise in antioxidant activity for these beans. For kidney beans and adzuki beans, the change in antioxidant activity may be related not only to the Maillard reaction but also to other factors affecting antioxidant activity.

### 3.5. Changes in the Resistant Starch Content of Beans

Resistant starch is not digested in the small intestine but is transported to the large intestine, where it is fermented by gut bacteria and plays a nutritional and physiological role similar to that of dietary fiber [36]. Studies on cooking methods have yielded mixed results regarding resistant starch content. One study found that boiling starchy beans reduced their resistant starch content [37], whereas another reported an increase with steaming [38]. Considering that a roasting temperature of 190 °C is significantly higher than the 100 °C typically used for boiling or steaming, we examined the effect of roasting on the resistant starch content. Peanuts, which are not classified as starchy beans, were excluded from this analysis.

**Figure 5:**
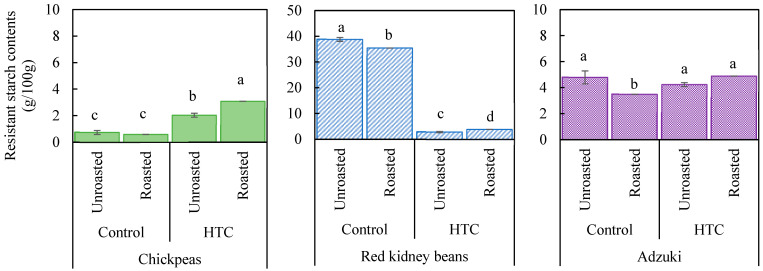
Resistant starch content of various beans as affected by storage conditions and roasting. Controls were stored at 4 °C and 80% relative humidity (RH) for 60 d; hard-to-cook (HTC) beans were stored at 30 °C and 75% RH for 60 d. Roasting was performed at 190 °C for 20 min. Significant differences within the same bean type are indicated by lowercase letters a, b, and c. *p* < 0.05 (n = 2).

The results showed that unroasted HTC chickpeas had a higher resistant starch content than the control (Figure 5). Furthermore, roasting significantly increased the resistant starch content of HTC chickpeas. These findings suggest that both storage and roasting may promote the formation of resistant starch in chickpeas. In contrast, a drastic decrease in the resistant starch content was observed for kidney beans stored under high-temperature and high-humidity conditions, and the effect of roasting was less pronounced compared to that for HTC chickpeas, although a small increase was noted. For adzuki beans, roasting significantly reduced the resistant starch content in the control, but no clear change was observed in the HTC beans.

These results suggest that in chickpeas, which initially have a low resistant starch content, the starch structure may have changed during storage under high-temperature and high-humidity conditions, promoting the formation of resistant starch. As shown in Figure 4, chickpeas have a high content of amylose, which can be converted into resistant starch. Additionally, heat treatments, such as roasting, may strengthen the interactions between starch and protein, further contributing to the increase in resistant starch [34]. In contrast, kidney beans showed a notably high resistant starch content in the control (38.9 g/100 g), which accounted for more than half of the total carbohydrate content (58.6%) (Table 1). In HTC kidney beans, the resistant starch content decreased to less than one-tenth of that in the control, and roasting resulted in only a minimal increase in that parameter. These results suggest that storing kidney beans under refrigeration is more effective at maintaining the resistant starch content than storing them under high-temperature and high-humidity conditions. For adzuki beans, neither storage nor roasting significantly affected the resistant starch content, likely owing to the greater stability of adzuki bean starch at high ambient temperatures compared with other beans [39].

Overall, these findings indicate that the effects of storage and roasting on the resistant starch content significantly vary among different beans, with distinct mechanisms at play for each. However, this study has some limitations. First, we analyzed a limited number of bean varieties, roasting conditions, and storage parameters, which may limit the generalizability of the findings. Second, the analytical methods used were unable to fully distinguish between the different forms of resistant starch, potentially influencing the accuracy of the results. Future studies addressing these limitations are needed to provide a more comprehensive understanding.

### 3.6. Potential and Synergistic Effects of HTC Storage and Roasting

Table 3 highlights the relative changes in the functional properties of legumes under HTC storage and roasting, normalized to unroasted control samples stored at 4 °C. Although HTC storage is often associated with reduced cooking quality, this table illustrates its potential to enhance certain functional properties, especially when combined with roasting. Values greater than 1.0 are shaded for emphasis.

Chickpeas, for instance, exhibit a significant synergistic effect, with antioxidant activity (DPPH) increasing dramatically and the resistant starch content showing a substantial improvement after both HTC storage and roasting. This suggests that HTC storage induces structural changes that prepare legumes for further functional enhancement during roasting, likely through the formation of bioactive compounds such as Maillard reaction products. In contrast, red kidney beans and adzuki beans display limited improvements, suggesting a need for alternative processing approaches to unlock their functional potential. Peanuts, conversely, showed moderate increases in antioxidant activity and polyphenol content after roasting, indicating that roasting can counteract some oxidative degradation caused by HTC storage.

The results presented in Table 3 underscore the potential to repurpose HTC beans, transforming them from a waste product into valuable functional food ingredients. Although the extent of these enhancements varies by legume type, the present study demonstrates that targeted processing strategies can maximize their functionality, aligning with global efforts to reduce food waste and develop sustainable food products.

## 4. Conclusions

In this study, we evaluated the impact of roasting on the antioxidant activity, reducing sugar content, and resistant starch content of various beans stored under high-temperature and high-humidity conditions (HTC beans). The results suggested that roasting has varying effects on the functional properties of different bean types. Both HTC chickpeas and peanuts showed a significant increase in antioxidant activity after roasting, whereas chickpeas showed an increase in the resistant starch content. In contrast, kidney beans and adzuki beans, which originally had high antioxidant capacity, showed only a slight increase in antioxidant activity after roasting. Thus, roasting HTC beans, such as chickpeas and peanuts, could enhance their antioxidant activity and other functional properties.

In conclusion, roasting offers a viable method for repurposing HTC legumes and reducing waste while enhancing their functional properties. Such an approach offers practical implications for the development of sustainable and health-oriented food products, catering to both industry needs and consumer preferences. This study provides a foundation for the effective exploitation of HTC beans by roasting, particularly when combined with powdering. Future research should perform component analyses to elucidate the mechanisms underlying such functional improvements induced by roasting.

## Figures and Tables

**Table 1:** General ingredients of various beans.

	Energy	Moisture	Protein	Lipid	Carbohydrate	Na
	kcal/100 g	g/100 g	g/100 g	g/100 g	g/100 g	mg/100 g
Chickpeas	366 ± 5.4	10.8 ± 0.02	19.2 ± 0.22	5.1 ± 0.14	62.3 ± 0.53	0
Red kidney beans	333 ± 6.1	14.8 ± 0.03	22.6 ± 0.14	1.5 ± 0.05	58.5 ± 0.16	0
Adzuki beans	344 ± 3.2	14.0 ± 0.07	20.9 ± 0.08	1.9 ± 0.12	60.1 ± 0.29	0
Peanuts	545 ± 12.7	6.1 ± 0.01	24.2 ± 0.09	47.3 ± 0.71	18.9 ± 0.36	0

**Table 2:** Color values of various beans as affected by storage conditions and roasting.

Bean Type	Storage	Roasting Status	L*	a*	b*	ΔE
Chickpeas	Control	Unroasted	85.6 ± 3.4	0.3 ± 1.0	26.4 ± 3.4	
		Roasted	81.3 ± 1.1	3.3 ± 0.2 *	29.9 ± 1.0	7.4 ± 0.8
	HTC	Unroasted	75.8 ± 17.8	5.1 ± 7.8	28.6 ± 4.2	5.0 ± 0.8
		Roasted	65.1 ± 3.0 *	9.4 ± 2.4 *	29.8 ± 2.8	22.9 ± 6.4
Red kidney beans	Control	Unroasted	82.3 ± 1.7	1.5 ± 0.8	11.2 ± 1.0	
		Roasted	73.2 ± 4.5 *	4.6 ± 1.3 *	23.7 ± 1.5 ***	16.1 ± 3.7
	HTC	Unroasted	80.9 ± 2.5	2.8 ± 1.1	15.0 ± 1.0 **	5.4 ± 1.0
		Roasted	67.7 ± 4.0 **	7.7 ± 2.5 *	27.6 ± 1.9 ***	22.9 ± 3.8
Adzuki	Control	Unroasted	80.6 ± 2.6	1.2 ± 0.2	10.8 ± 1.3	
		Roasted	65.3 ± 0.7 **	7.2 ± 0.3 ***	29.0 ± 0.2 *	24.7 ± 0.8
	HTC	Unroasted	75.6 ± 2.5 *	4.0 ± 1.6 *	14.0 ± 2.1	6.5 ± 2.5
		Roasted	65.4 ± 4.4 *	9.2 ± 0.8 ***	26.6 ± 1.6 **	23.5 ± 2.3
Peanut	Control	Unroasted	67.2 ± 0.0	6.7 ± 1.2	18.4 ± 1.5	
		Roasted	37.6 ± 6.9 **	11.3 ± 4.5	16.2 ± 9.7	27.8 ± 4.6
	HTC	Unroasted	63.8 ± 2.5	8.8 ± 2.0	22.2 ± 6.2	12.3 ± 0.6
		Roasted	39.7 ± 13.0 *	15.1 ± 0.8 **	24.6 ± 2.4 **	18.0 ± 11.0

Controls were stored at 4 °C and 80% relative humidity (RH) for 60 d; hard-to-cook (HTC) beans were stored at 30 °C and 75% RH for 60 d. Roasting was performed at 190 °C for 20 min. L* represents brightness ranging from 0 (black) to 100 (white); a* indicates the degree of redness (+a*) or greenness (−a*); and b* indicates yellowness (+b*) to blueness (−b*). The color change (ΔE) was calculated as the difference in color values relative to the unroasted beans stored at 4 °C and 80% RH. Values are indicated as the mean ± standard deviation (*n* = 6). * *p* < 0.05, ** *p* < 0.01, and *** *p* < 0.001 compared with the unroasted group stored at 4 °C and 80% RH.

**Table 3:** Relative changes in functional properties of legumes after hard-to-cook storage and roasting.

			DPPH	ORAC	Total Polyphenol Content	Reducing Sugar	Resistant Starch	L*	ΔE
Chickpeas	Control	Unroasted	1.0	1.0	1.0	1.0	1.0	1.0	
		Roasted	2.5	1.0	0.8	0.6	0.8	1.0	7.4
	HTC	Unroasted	4.0	1.1	1.0	2.8	2.7	0.9	5.0
		Roasted	13.8	1.2	1.4	0.8	4.1	0.8	22.9
Red kidney beans	Control	Unroasted	1.0	1.0	1.0	1.0	1.0	1.0	
		Roasted	0.9	0.8	0.9	0.4	0.9	0.9	16.1
	HTC	Unroasted	0.8	1.0	0.8	1.0	0.1	1.0	5.4
		Roasted	0.8	0.8	0.9	0.5	0.1	0.8	22.9
Adzuki	Control	Unroasted	1.0	1.0	1.0	1.0	1.0	1.0	
		Roasted	0.7	0.9	0.8	0.6	0.7	0.8	24.7
	HTC	Unroasted	0.6	0.7	0.6	0.9	0.9	0.9	6.5
		Roasted	0.5	0.6	0.5	0.4	1.0	0.8	23.5
Peanut	Control	Unroasted	1.0	1.0	1.0	1.0		1.0	
		Roasted	1.3	0.9	1.3	0.4		0.6	27.8
	HTC	Unroasted	0.8	0.6	1.1	1.2		1.1	12.3
		Roasted	1.1	0.7	1.5	0.4		0.7	18.0

Values greater than 1.0 are shaded for emphasis.

## Data Availability

The original contributions presented in this study are included in the article. Further inquiries can be directed to the corresponding author.
